# State of the ART: Drug Screening Reveals Artesunate as a Promising Anti-Fibrosis Therapy

**DOI:** 10.70322/jrbtm.2024.10023

**Published:** 2024-12-16

**Authors:** Yujie Qiao, Jiurong Liang, Dianhua Jiang

**Affiliations:** 1 Division of Pulmonary, Women’s Guild Lung Institute, Department of Medicine, Los Angeles, CA 90048, USA; 2 Department of Respiratory and Critical Care Medicine, Nanfang Hospital, Southern Medical University, Guangzhou 510515, China; 3 Department of Biomedical Sciences, Cedars-Sinai Medical Center, Los Angeles, CA 90048, USA

**Keywords:** Fibrosis, Drug screening, Artesunate, MD2/TLR4

## Abstract

Fibrosis is a progressive pathological process that severely impairs normal organ function. Current treatments for fibrosis are extremely limited, with no curative approaches available. In a recent article published in *Cell*, Zhang and colleagues employed drug screening using ACTA2 reporter iPSC-derived cardiac fibroblasts and identified artesunate as a potent antifibrotic drug by targeting MD2/TLR4 signaling. This study provides new insights into strategies for exploiting existing drugs to treat fibrosis.

## Main Text

Fibrosis is an excessive accumulation of extracellular matrix (ECM) resulting from an aberrant wound healing response caused by repeated injury, which can lead to distortion of tissue architecture and loss of organ function. Fibrosis can occur in any solid organ and tissue, resulting in the failure of various vital organs, including the lung, heart, liver and kidney [1–3]. Despite its severe symptoms and poor prognosis, therapies for organ fibrosis remain limited for decades. Only two drugs, pirfenidone and nintedanib, have been approved for treating idiopathic pulmonary fibrosis (IPF), neither of which, however, can fully halt disease progression, and both have significant adverse effects [4,5]. Therefore, there is an urgent need to develop more strategies to target organ fibrosis. In recent years, phenotypic drug discovery (PDD) has attracted immense attention in drug discovery. Compared with traditional target-based drug discovery (TDD), PDD focuses on the effects of the potential drug targets on disease pathophysiology instead of on the specific molecular or chemical structures, demonstrating significantly higher efficiency in pharmaceutical industry productivity [6,7]. Meanwhile, emerging technologies such as high-throughput screening (HTS), high-content screening (HCS), and in silico simulations provide practical support for the application of PDD [8–10]. This strategy is highly valuable in addressing the gap for discovering more potential antifibrotic drugs.

A recent study by Zhang et al. published in the journal *Cell* employed a drug screening system and identified artesunate (ART) as a promising anti-cardiac fibrosis compound [11]. To solve the problem that human primary cardiac fibroblasts (CFs) are insufficient for large-scale drug screening and replicates, the authors developed induced pluripotent stem cell (iPSC) derived human cardiac fibroblasts (iPSC-CFs), which remain quiescent up to P5 and serve as an ideal cell source for high-throughput screening (HTS). To enable real-time monitoring of myofibroblast activation and quantify the effectiveness of screened compounds, the authors generated an iPSC reporter cell line expressing Clover2 from the ACTA2 locus, a key myofibroblast marker gene, using CRISPR-Cas9. Then, the ACTA2^Clover2^ iPSC-CFs were generated in accordance with cardiac fibroblast development. Initially, the ACTA2^Clover2^ iPSC-CFs were stimulated with TGF-β1 and treated with approximately 5000 candidate compounds from a library of Known Bioactives and FDA Approved Drugs, followed by live cell Hoechst staining. High-content confocal microscope was then used to capture the Clover2 and Hoechst signals, generating 7-point dose-response curves and calculating half maximal effective concentration (EC_50_) and toxic concentration 50 (TC_50_) values. Compounds with high EC_50_ and low TC_50_ were excluded. The remaining candidates were further filtered using a machine learning predictor, yielding 20 compounds. After toxicity assessments using iPSC derived cardiomyocytes (iPSC-CMs) and iPSC derived endothelial cells (iPSC-ECs), the top hit was selected and used to perform *in vitro* and *in vivo* functional tests. Finally, the antifibrotic mechanism of the selected compound was investigated. Through the screening, the authors found artesunate (ART), a classic anti-malaria drug [12], emerged as a top candidate with a relatively low EC_50_ of 2.1 μM without toxicity to iPSC-CMs or iPSC-ECs up to 10 μM.

The authors next conducted a series of *in vitro* and *in vivo* experiments to evaluate the antifibrotic effects of ART. In 2D *in vitro* studies, ART treatment inhibited TGF-β1 induced fibrotic gene expression, cell proliferation, migration, gel contraction, and collagen secretion from CFs. IPSC-derived 3D-engineered heart tissue models demonstrated significant improvement in contraction and relaxation velocity, alleviation of passive tissue tension, and suppression of fibrotic gene expression following ART treatment. *In vivo*, preventive or therapeutic TAC models, as well as an ischemia-reperfusion model, were utilized to mimic preclinical and clinical scenarios. ART was effective in attenuating fibrosis and improving cardiac function with the *in vivo* models. Single-nucleus RNA sequencing (snRNA-seq) revealed that ART inhibited TAC-induced dynamic transformation in CFs.

**Figure 1.**
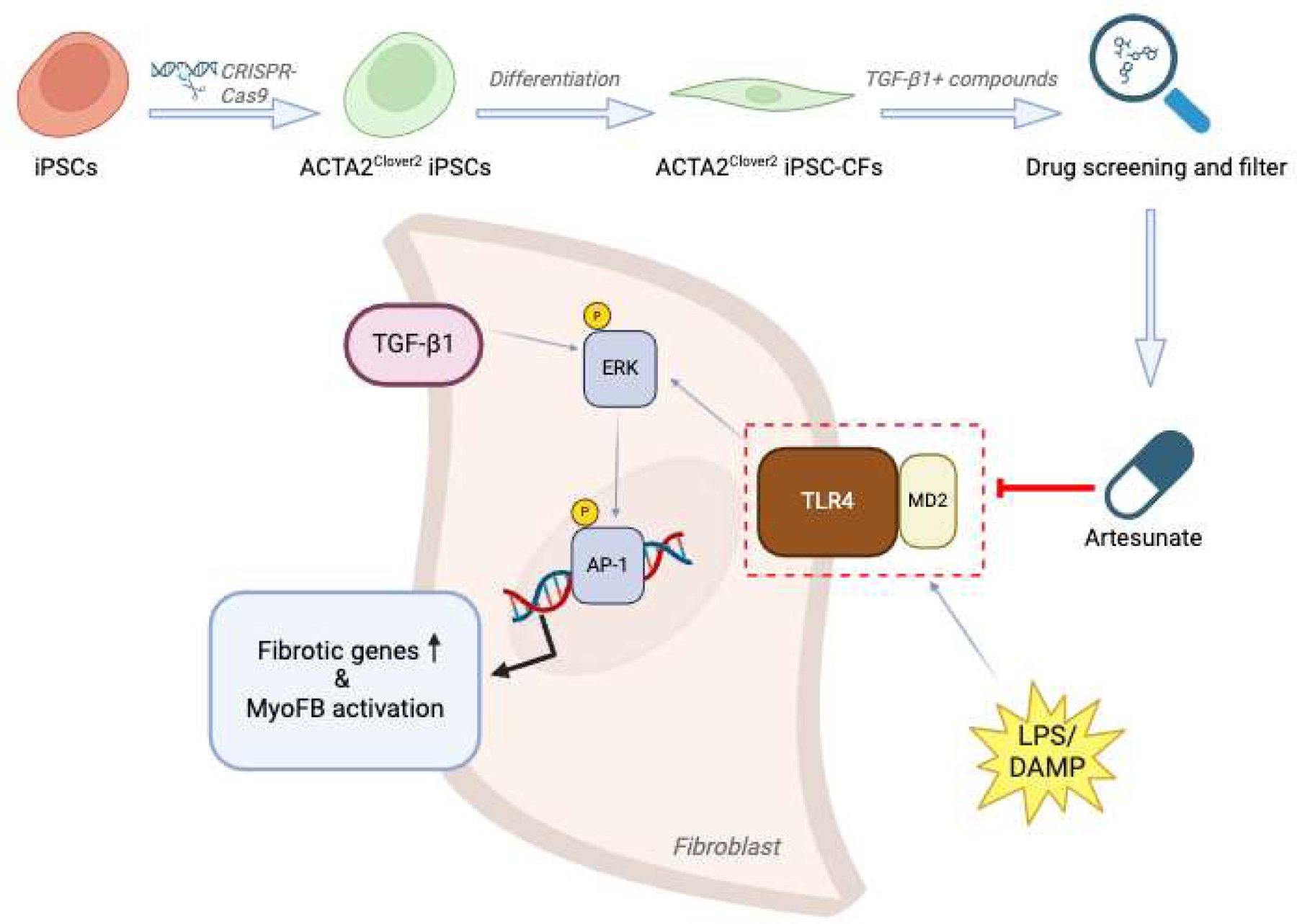
A schematic diagram illustrating the study workflow and the functional pathway of ART in CFs. ACTA2^Clover2^ iPSC-CFs were differentiated from ACTA2^Clover2^ iPSCs, which were generated using CRISPR-Cas9. Drug screening was conducted following TGF-β1 stimulation and treatment with candidate compounds, leading to the identification of artesunate as a potent antifibrotic drug. Artesunate inhibited MD2/TLR4 signaling activated by DAMPs, thereby suppressing ERK activation—a noncanonical TGF-β pathway—and the subsequent AP-1 pathway, ultimately reducing fibrotic genes transcription and preventing myofibroblast activation. The diagram was generated with BioRender (https://www.biorender.com/).

ART is well known for its role in malaria therapy by binding heme [13]. However, heme-related genes were absent in cardiac fibroblasts. The authors then investigated the role of ART in inhibiting MD2/TLR4 signaling. Surface plasmon resonance (SPR) assay confirmed the binding between ART and MD2. In silico simulations suggested that ART reduced the flexibility of the TLR4 binding domain in MD2, thus antagonizing MD2-TLR4 interaction. MD2 conformational change was further validated by intrinsic tryptophan fluorescence at Tryptophan 23, which was transitioned from a buried hydrophobic state to a solvent-exposed position upon ART binding. Additionally, the ability of ART to interfere with LPS-MD2 binding was also evaluated, showing a stronger competitive binding to LPS compared with L6H21, which is another MD2-TLR4 inhibitor that interferes with the interaction between LPS and MD2. Proximity ligation assay (PLA) and co-immunoprecipitation (coIP) further verified that MD2-TLR4 interaction was inhibited by ART. ERK signaling, one of the major downstream pathways of MD2/TLR4, was also inhibited by ART. Transposase-accessible chromatin (ATAC)-seq indicated AP-1, especially c-FOS, was the top downregulated transcription factor by ART upon TGF-β1 stimulation. In summary, this study demonstrated that ART inhibited MD2-TLR4 interaction by targeting MD2 molecules, thereby suppressing ERK and AP-1 signaling to reduce fibrotic gene expression in cardiac fibroblasts (Figure 1).

This study employs several state-of-the-art approaches to discover ART as an anti-fibrotic agent. Cell availability poses a significant challenge in anti-fibrosis drug screening due to the limited quantity and unsustainability of primary cells. The authors developed iPSC derived cells as the cell model, addressing the issue of cell quantity while preserving the features of primary cells. ACTA2^Clover2^ reporter cells were further generated to screen out potent antifibrotic compounds. This approach significantly increased the sensitivity and efficiency of drug screening, as well as the convenience of successive functional tests. The candidate compounds used in the screening were from libraries of Known Bioactives and FDA Approved Drugs [14], which are typically clinically established drugs repurposed for new applications rather than newly developed drugs. This approach of repurposing clinically established drugs for new applications significantly reduced time and resource costs. For functional tests, the authors utilized a range of *in vitro* and *in vivo* models, including a 2D cell model, a 3D engineered heart tissue model, and three *in vivo* models that mimic cardiac hypertrophy and myocardial infarction in clinical settings. The results strongly supported the therapeutic potential of ART in treating cardiac fibrosis. The in silico simulations and sequencing data provided insights into the potential mechanism of ART in treating fibrosis, which involves ART binding to MD2 to inhibit TLR4 signaling and its cascades. We previously reported that TLR4 interacted with hyaluronan to participate in the regulation of lung inflammation and injury [15], while also playing a critical role in the renewal of type 2 alveolar epithelial cells and limiting lung fibrosis [16]. TLR4 was also reported to promote fibrogenesis by activating quiescent hepatic stellate cells (HSCs) [17]. These studies collectively emphasized the crucial role of TLR4 in the pathogenesis of fibrosis. As the authors cited in the article, among all TLR family members, only TLR4 requires MD2 as a co-receptor [18], highlighting the link between specific inhibition of MD2/TLR4 signaling by ART and its therapeutic potential in organ fibrosis. Additionally, ERK, a critical factor in myofibroblast activation and lung fibrosis [19], was investigated to be suppressed by ART, further implicating its effectiveness in anti-fibrosis. This study also underscores the endless new applications of artemisinins [20,21].

While the study was comprehensive and well-detailed, some points remain elusive. The mechanistic link between ART-MD2 and TGF-β signaling was unclear. Furthermore, other pathways beyond TGF-β signaling may also be involved in ART’s antifibrotic effects. In addition, while LPS is the most well-known activator of the TLR4 pathway [22,23], it is not a common inducer in fibrosis, especially in non-infectious cardiac injury. Additional damage-associated molecular patterns (DAMPs), such as hyaluronan [15,16] and the nuclear protein high-mobility group box 1 (HMGB1) [24,25], which are more relevant to non-infectious tissue fibrosis, should be considered for molecular interaction studies. Moreover, the long-term effects and safety of ART, and comprehensive investigations into its potential in other organ fibrosis, remain further to be evaluated.
